# Effects of Collagen Hydrolysate-Based Protein Complexes on Physical Endurance, Glucose Metabolism, and Renal Function in Rats

**DOI:** 10.3390/nu18111735

**Published:** 2026-05-28

**Authors:** Denis V. Kurkin, Dmitry A. Bakulin, Nazar A. Osadchenko, Natalia S. Murina, Elena V. Litvinova

**Affiliations:** 1 Department of Pharmacy, Russian University of Medicine, Moscow 119049, Russia; strannik986@mail.ru (D.V.K.);; 2 Department of Technology and Biotechnology of Meat and Meat Products, Russian Biotechnology University, Moscow 125080, Russia; murinans@mgupp.ru (N.S.M.);

**Keywords:** collagen hydrolysate, protein supplementation, physical endurance, glucose metabolism, renal function, high-protein diet, functional food ingredient, Wistar rats

## Abstract

Background/Objectives: The increasing prevalence of nutrition-related diseases and the limited availability of convenient, metabolically safe, high-protein foods represent a pressing public health challenge. This study aimed to evaluate the effects of four composite animal-derived high-protein ingredients based on collagen enzymatic hydrolysates on physical endurance, feeding behaviour, carbohydrate metabolism, renal function, and behavioural parameters in rats. Methods: Four lyophilised collagen hydrolysate-based ingredients were developed using enzymatic biotransformation of bovine and porcine raw materials, combined with bovine whey protein concentrate, bovine meat trim hydrolysate, porcine blood plasma proteins, and an api-component (Samples 1–4; protein content 87–89%). Ninety male Wistar rats were randomised into one control group and four experimental groups (n = 20 per experimental group, n = 10 controls) and received test samples by intragastric gavage at 3000 mg/kg/day for 40 days. Physical endurance was assessed via a weighted forced swimming test (days 0, 30, and 40); behavioural status by open field, adhesive removal, and marble burying tests; and biochemical parameters (blood glucose, serum urea, creatinine, urinary protein, and GFR) at days 0 and 40. Results: All experimental groups demonstrated a significant reduction in standard chow consumption (19–24%, *p* < 0.01) without affecting body weight gain. Physical endurance improved significantly in all groups relative to baseline, with the most pronounced effect in the Sample 3 group (+39% at day 40, *p* < 0.05). Blood glucose levels were significantly reduced across all groups (9–16%, *p* < 0.05). No adverse behavioural effects were observed. Biochemical markers indicated an adaptive rather than pathological renal response, with elevated GFR in three of four experimental groups (*p* < 0.05) and reduced proteinuria in the Sample 1 and Sample 3 groups. Conclusions: Forty-day administration of collagen hydrolysate-based protein complexes improved physical endurance and glucose metabolism, reduced food intake without compromising body weight, and did not impair renal function or behavioural status in healthy adult rats. These findings support the potential of such ingredients as functional food components, pending confirmation of long-term safety in extended studies.

## 1. Introduction

The burden of nutrition-related diseases, including obesity, type 2 diabetes mellitus, and metabolic dysfunction-associated steatotic liver disease, has continued to increase over recent decades, posing a major global medical and social challenge. Despite substantial advances in understanding the pathophysiology of these conditions, prospects for effective control of this epidemic remain unfavorable. Lifestyle modification and current pharmacological therapies remain accessible to only a limited proportion of patients because of behavioral, economic, and organizational barriers [1,2].

The problem of unhealthy dietary patterns in a substantial proportion of the population is systemic in nature and is driven by multiple factors. Among the most important of these are eating habits established early in life. Their correction in later adulthood, when nutrition-related diseases often emerge against a background of reduced metabolic adaptability, remains highly challenging [3,4]. Another major difficulty is the limited availability of convenience foods that are not only safe in terms of their long-term health effects but also possess appealing sensory properties. A paradox of contemporary eating patterns is that, despite food abundance, the primary concern shifts toward long-term metabolic safety under conditions of chronic excess intake of calories and nutrients, that is, consumption exceeding energy and physiological requirements [5].

Modern technologies, including those applied in agriculture and food processing, have radically transformed the food environment by providing unprecedented diversity and accessibility of food products. However, the range of ready-to-eat convenience foods with long-term metabolic safety that can compete with high-calorie alternatives associated with an increased risk of adverse health outcomes remains limited. Widely available and affordable convenience foods designed for rapid energy replenishment often contain excessive amounts of calories, fats, and flavor enhancers, which promotes their habitual rather than occasional consumption beyond recommended limits [5].

Under contemporary conditions, the properties that determine the consumer appeal of food products are often shaped by manufacturers’ marketing strategies rather than by the actual public health needs of society. This creates an objective conflict between societal interests aimed at preserving health and work capacity and the economic interests of manufacturers. A balance is therefore required in which population health, as well as quality and longevity of life, remains the priority. It is important to note that the concept of “naturalness” is not synonymous with “health benefits”, since many natural components, including salt, sugar, and saturated fats, may pose health risks when consumed in excess. In this context, the development of products containing highly digestible protein and comparable in price and sensory properties to popular alternatives is of considerable scientific and practical relevance. Key requirements for such products include alignment with consumer expectations, a wide safe consumption range, affordability, and demonstrated health benefits. Among available protein sources, animal-derived proteins are distinguished by a complete amino acid profile and high bioavailability [6].

Thus, the development of new protein products that can compete with tasty, high-calorie analogues while remaining safe for consistent consumption by different population groups is a pressing scientific and practical task.

Collagen hydrolysates have attracted growing interest as functional food ingredients due to their distinctive technological properties, wide availability of raw materials, and accumulating evidence of physiological activity. Unlike intact collagen, enzymatic hydrolysates yield soluble peptide fractions with high digestibility and demonstrated bioactivity in preclinical and clinical settings, including effects on musculoskeletal remodelling, glucose metabolism, and satiety [7,8]. However, the majority of published studies have examined isolated collagen peptides as single-ingredient supplements. Multi-component formulations combining collagen hydrolysates with complementary animal-derived protein sources—such as whey protein concentrate, meat hydrolysates, and porcine blood plasma proteins—represent a distinct category of ingredient with a broader amino acid and peptide profile, yet remain substantially understudied in terms of their combined physiological effects. In particular, no preclinical studies have comprehensively evaluated such multi-component collagen-based blends with respect to physical endurance, glucose metabolism, renal function, and behavioural parameters simultaneously, which are all relevant endpoints for assessing the safety and functional potential of candidate food ingredients.

This study aimed to evaluate the physiological effects of four novel multi-component high-protein ingredients based on enzymatic collagen hydrolysates—formulated in combination with bovine whey protein concentrate (a bovine milk-derived protein fraction obtained by ultrafiltration following casein removal), meat trim hydrolysate, porcine blood plasma proteins, and an api-component—on physical endurance, feeding behaviour, carbohydrate metabolism, renal function, and behavioural parameters in rats, and to assess their potential as functional food ingredients.

## 2. Materials and Methods

### 2.1. Collagen Processing

The study materials consisted of composite high-protein ingredients based on hydrolyzed collagen. A traditional source of collagen and its hydrolysates is the connective tissue of farm animals, primarily cattle and pigs. Native collagen is insoluble in water, capable of swelling at pH 5–7, and characterized by an isoelectric point within the pH range of 6.0–7.0. The pH was maintained using Tris-HCl buffer. The protein is thermolabile: at 63–64 °C, collagen fibers undergo deformation and contraction, whereas temperatures above 70 °C induce pronounced degradation with the formation of gelatin fractions (gelatoses); typically, 20–45% of collagen is converted into glutin. In its native form, collagen is poorly absorbed. An important factor underlying the relevance of collagen use in food products is its high content of glycine and proline, together with the absence of tryptophan, which confers specific functional properties.

To enhance the functionality and digestibility of collagen-containing raw materials, structural modification approaches are employed to induce varying degrees of disruption of the native protein conformation, up to the formation of peptides and free amino acids, including alkaline-salt, acidic, thermal, and enzymatic treatments. Collagen is a polymer, whereas its hydrolysis products are polyampholytes characterized by the presence of an isoelectric point.

Controlled hydrolysis enables the production of hydrolysates with defined molecular weight, which in turn determines the functional properties of the resulting food ingredients. This aspect is critical, as di- and tripeptides are generally considered to be absorbed more efficiently; however, such hydrolysates often exhibit limited processability while still allowing the development of functional food products. The choice of transformation approach and processing conditions enables selective cleavage of specific peptide bonds, resulting in hydrolysates with distinct properties.

Among the available approaches for the modification of collagen-containing raw materials, biotransformation was selected as the most promising strategy, involving the choice of collagenolytic enzymes, optimization of processing conditions, and adaptation of the technology to the specific type of collagen substrate. In the present study, this approach was used to obtain collagen enzymatic hydrolysates.

**Figure 1:**
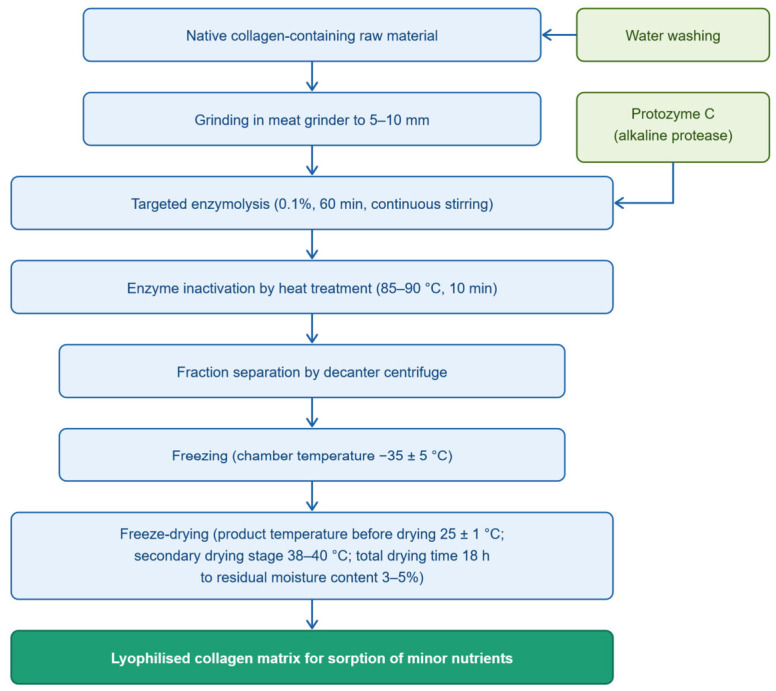
General scheme of enzymolysis of collagen-containing raw materials.

Experimental procedures were performed under standard laboratory conditions (temperature 20 ± 2 °C, relative humidity 40–60%). The overall workflow of collagen-containing raw material enzymolysis is presented in Figure 1.

To obtain hydrolyzed collagen, porcine and bovine raw materials were placed in the reaction medium and subjected to preliminary preparation. Raw material preparation involved grinding porcine skin and bovine by-products (lips, rumen, and lungs) in a meat grinder fitted with a perforated plate (5–10 mm aperture) to increase the surface area available for enzyme contact. Prior to enzyme addition, the reaction buffer was prepared. The required buffer volume was calculated based on a solid-to-liquid ratio of 1:2, as this proportion ensures the formation of a flowable suspension.

Purified (distilled) water was used as the solvent; alternatively, potable water meeting standard quality requirements may be applied. Insufficient water purity can lead to reduced enzyme activity, incomplete hydrolysis, and the formation of products with unstable quality. The presence of heavy metal ions may inhibit enzymatic activity through interactions with the active site, whereas chlorine and other oxidizing agents can induce denaturation of protein molecules, including enzymes. In addition, extraneous salts may disrupt the buffering capacity of the reaction medium.

Collagen-containing raw materials were enzymatically processed using the enzyme preparation Protozyme C (alkaline protease). Protozyme C is a fungal alkaline protease derived from a selected strain of *Acremonium chrysogenum*, produced by LLC Trading House Biopreparate (Russia). The optimal activity of the enzyme preparation was observed at pH 7.0, while the temperature of the reaction medium was maintained at 23 ± 1 °C. This temperature range was selected to ensure an optimal enzymatic reaction rate while preserving enzyme stability.

The prepared raw materials, water, and enzyme solution were simultaneously loaded into a thermostatically controlled stirred reactor. The enzyme preparation was added as a solution at 0.1% of the raw material mass to ensure uniform distribution. Enzymolysis was carried out for 60 min under continuous stirring with strict temperature control and pH monitoring. After 60 min, the reaction mass was heated to 85–90 °C and held for 10 min to inactivate the enzyme preparation. The hot mass was then transferred to a decanter centrifuge to separate the liquid fraction from the wet pellet (target product). The wet pellet was spread as a thin layer (1–2 cm) onto freeze-dryer trays and subjected to deep freezing at −40 °C. The samples were then lyophilized during the primary drying stage under low vacuum pressure (0.1–0.3 mbar). Upon completion of sublimation, the temperature was gradually increased to 25–30 °C while maintaining the vacuum to remove residual moisture.

**Figure 2:**
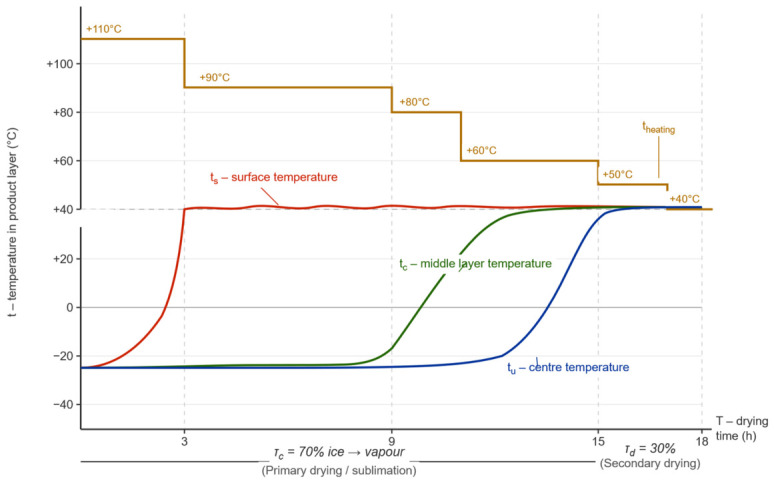
Freeze-drying of collagen lysates.

The lyophilization profile of the collagen enzymatic hydrolysates is shown in Figure 2.

The desorption stage was considered complete when the residual moisture content of the final product reached 3–5%. The process yielded approximately 21.5 kg of lyophilized collagen hydrolysate per 100 kg of initial raw material on a dry matter basis.

The lyophilized collagen enzymatic hydrolysates were stored under low-humidity conditions in sealed containers protected from natural light. Lyophilization was selected over frozen storage to extend shelf life and ensure compatibility with the dry blending process, as all components of the final formulations contained no more than 5% moisture.

**Figure A1:**
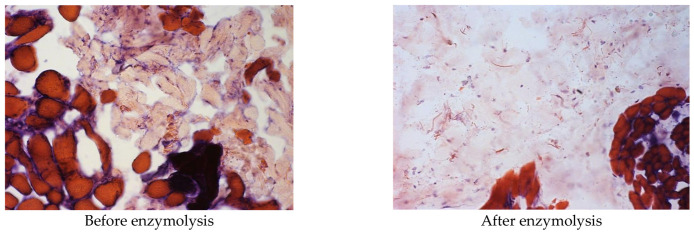
Microstructure of bovine lips before and after controlled enzymatic modification (objective magnification ×20).

Evidence of collagen-containing raw material disruption was provided by histological analysis. Representative histological sections of the enzymatic hydrolysates before and after targeted modification are shown in Figure A1. Histological examinations were performed according to established methodological requirements. Sample sections with a thickness of 16 μm were prepared using a Microm HM525 cryostat (Thermo Scientific Walldorf, Germany) and stained with Ehrlich’s hematoxylin and a 1% aqueous-alcoholic eosin solution (BioVitrum, Moscow, Russia). Histological specimens were examined and photographed using an AxioImager A1 light microscope (Carl Zeiss, Oberkochen, Germany) equipped with an AxioCam MRc 5 camera. Image processing was performed using the AxioVision 4.7.1.0 image analysis system adapted for histological studies.

### 2.2. Physicochemical and Nutritional Properties of Test Materials

Collagen hydrolysate-based blends generally offer several advantages: they immobilise active components with subsequent sustained release, protect incorporated substances from degradation, and exert a regenerative effect.

Microstructural examination of sections obtained from untreated bovine lip tissue demonstrated a clearly distinguishable fibrous architecture in the control samples. Following enzymatic treatment, further loosening, disintegration, and fragmentation of the fibers were observed, together with fiber thinning and the formation of a more homogeneous extracellular matrix.

To develop amino acid-balanced high-protein ingredients based on hydrolyzed collagen, a formulation was prepared consisting of dried collagen hydrolysate, api-component (bee pollen as a natural source of ascorbic acid), porcine blood plasma proteins, and bovine whey protein concentrate as sources of complete proteins. The selected ingredients were blended to homogeneity using a dry powder mixer. The key formulation requirements included component uniformity and controlled particle size. Component proportions were determined using a mathematical optimization model, with nutritional and biological value as the primary criteria, with particular emphasis on the recommended ratio of branched-chain amino acids (BCAAs).

The suitability of the test samples for dietary use was assessed based on their chemical and nutrient composition, functional-technological properties, and in vitro biological evaluation. Standardized analytical methods were used to determine nutritional value parameters, including moisture, protein, fat, and ash content. Carbohydrate content was calculated by difference, and organoleptic properties were evaluated using a standardized sensory assessment protocol.

Water-holding capacity and fat-holding capacity were determined according to the method of Lipatov Jr. [9] (see Appendix B for detailed protocols). Water-binding capacity was assessed by the pressing method. Yield stress was calculated from the mean penetration depth values.

Biological value was assessed using the calculation-based Lipatov method [9] with determination of amino acid score (%), the coefficient of amino acid score difference (%), potential biological value (%), the protein utility coefficient (R_C_, relative units), and the comparable redundancy coefficient (α, g).

In vitro protein digestibility was assessed using the Pokrovsky–Ertanov method [9] (see Appendix B for detailed protocol) through sequential exposure of sample proteins to a protease system consisting of pepsin and trypsin; hydrolysis products were quantified by the Lowry colorimetric reaction on a spectrophotometer and expressed as mg tyrosine per g protein.

The mass concentrations of amino acids, vitamins, and other nutrients were determined by high-performance liquid chromatography (HPLC) using a Milichrom A-02 system equipped with a stainless-steel chromatographic column packed with a reversed-phase C18 sorbent.

### 2.3. Animals

The experimental study was conducted using 90 sexually mature male Wistar rats aged 5 months and weighing 240–260 g. The animals were obtained from the breeding facility of the Scientific Center for Biomedical Technologies, Federal Medical-Biological Agency of Russia (Andreevka branch). All procedures were performed in accordance with Directive 2010/63/EU and were approved by the Interuniversity Ethics Committee (Protocol No. 03, dated 13 March 2025).

Animals were housed in groups of five in polypropylene cages (type T4B, MEST LLC, Moscow, Russia; 545 × 395 × 200 mm) with combined bedding (wood pellets and soft shavings), under a 12 h/12 h light/dark cycle with air exchange of 14–15 volumes per hour. Temperature was maintained at 20–26 °C with relative humidity of 30–70%. A standard pelleted rodent diet (GOST R 51849-2001) was provided *ad libitum*. Purified drinking water meeting SanPiN 2.1.4.1074-01 requirements was provided *ad libitum* and refreshed every 24 h. Following delivery, all animals underwent a 14-day veterinary quarantine and acclimatisation period before experimental procedures commenced.

At the end of the experiment, animals were euthanised by exposure to CO_2_ in a dedicated inhalation chamber, in accordance with Directive 2010/63/EU. This method was selected to minimise pain, suffering, and distress and was performed by trained personnel. No animals were removed from the study prior to its completion, and no unexpected adverse events were observed during the experimental period. Daily clinical observations served as the primary monitoring tool; no formal humane endpoints requiring early termination were triggered.

### 2.4. Experimental Design

Four composite high-protein ingredients based on hydrolyzed collagen were prepared as described in Section 2.1 and used as test samples. All samples were crystalline powders ranging in colour from white to light cream, with no characteristic odour, and had a molecular weight not exceeding 12 kDa. The composition of each sample was as follows:

Sample 1: Bovine collagen hydrolysate: bovine whey protein concentrate = 50:40.

Sample 2: Porcine collagen hydrolysate: bovine whey protein concentrate = 50:40.

Sample 3: Bovine collagen hydrolysate: bovine meat trim hydrolysate: porcine blood plasma proteins: api-component = 50:40:5:5.

Sample 4: Porcine collagen hydrolysate: bovine meat trim hydrolysate: porcine blood plasma proteins: api-component = 50:40:5:5.

Following a 14-day quarantine and acclimatization period, the rats were assigned by stratified randomization into five groups: one control group (n = 10) and four experimental groups (n = 20 per group).

**Figure 3:**
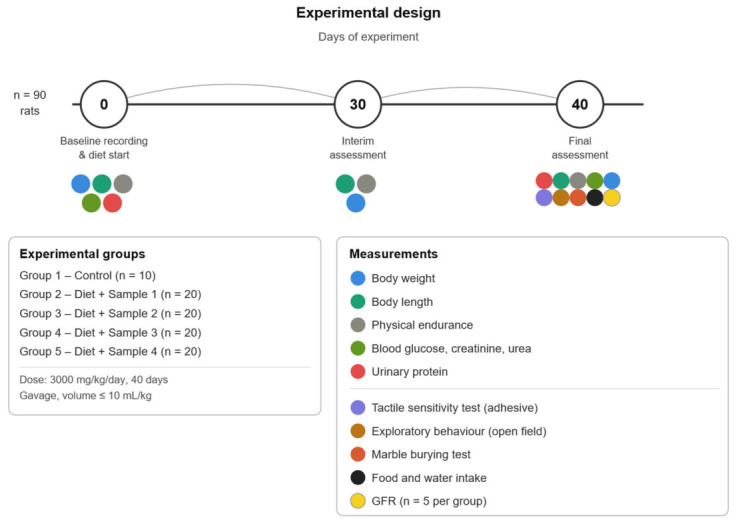
Experimental design.

For 40 days, the animals received the test substances once daily by intragastric gavage (Figure 3):

The control group received the standard diet alone.

Animals in the experimental groups additionally received one of the four developed composite ingredients: sample 1 (89% protein), sample 2 (88% protein), sample 3 (87% protein), or sample 4 (88% protein) at a dose of 3000 mg/kg/day.

The sample size was determined based on prior laboratory experience with the variability of the test systems employed, ethical considerations, and the expected variability of the outcome data; no formal *a priori* power calculation was performed. The study was not blinded: group allocation was known to all personnel involved in animal husbandry, sample administration, and outcome assessment, as blinding was not feasible given the nature of the gavage administration protocol.

The administration volume did not exceed 10 mL/kg. The dose was adjusted according to body weight, which was measured every 10 days.

### 2.5. Outcome Measures

To comprehensively evaluate the effects of the protein supplements, the following parameters were monitored:

1. Physiological status. Daily observations included behavior, coat condition, and mucous membrane appearance. Body weight was measured regularly, and daily food and water intake were recorded.
2. Physical endurance. Endurance was assessed using a forced swimming test with an additional load corresponding to 10% of body weight until exhaustion on days 0, 30, and 40 of the experiment [10].
3. Behavioral tests (performed on day 40):
  - Open field test: Assessment of locomotor and exploratory activity [11].
  - Adhesive removal test: Assessment of sensorimotor function based on the latency to detect and remove a sticker from the paw [12].
  - Marble burying test: Assessment of anxiety-like and compulsive-like behavior [13].
4. Biochemical markers (days 0 and 40) [14]:
  - Blood: Serum glucose levels were measured using a OneTouch UltraEasy glucometer (Malvern, PA, United States); serum urea was determined by the urease–phenol–hypochlorite method [15], and creatinine by the Jaffe method [16].
  - Urine: Urinary protein levels were measured using the pyrogallol red method [17]. Additionally, in five animals from each group, daily diuresis and urinary creatinine levels were assessed [16].

Glomerular filtration rate (GFR) was assessed in a randomly selected subset of 5 animals per group due to the technical demands of simultaneous 24 h urine collection and blood sampling. This sample size is consistent with established protocols for creatinine clearance studies in rodents [18,19] and provides adequate statistical power for detecting physiologically relevant changes in renal function.

### 2.6. Statistics

Statistical analysis was performed using GraphPad Prism 10 (GraphPad Software Inc., USA) and Microsoft Excel. Normality of data distribution was assessed using the Shapiro–Wilk test. Comparisons among multiple groups were performed using one-way analysis of variance (ANOVA), followed by Dunnett’s post hoc test for comparisons of experimental groups versus the control group. Data are presented as mean ± standard error of the mean (SEM). Differences were considered statistically significant at *p* < 0.05. For variables that deviated from normal distribution, box-and-whisker plots were supplemented with individual data points to improve visualization of data dispersion and group-level variability.

## 3. Results

### 3.1. Structural and Nutritional Characterization of Composite Ingredients

The physicochemical, nutritional, and amino acid composition of the four developed composite ingredients were assessed using standardized methods described in Section 2.2. The functional and technological properties are summarized in Table 1, the chemical and nutrient composition—including protein content, micronutrients, and in vitro digestibility—in Table 2, and the amino acid profile with biological value indices in Table 3.

The obtained results showed that the developed composite ingredients were characterized by a balanced chemical composition and contained ascorbic acid, which may support the repair of connective tissue proteins, primarily collagen, thereby contributing to the maintenance of the organism’s adaptive capacity. An important aspect of the analysis was the comparative evaluation of the component composition to ensure a balanced protein profile. Samples 1 and 2 contained bovine whey protein concentrate, which, in addition to harmonizing the amino acid composition, is associated with relatively high allergenicity, potentially limiting its use in sports nutrition. In contrast, samples 3 and 4 contained porcine blood plasma proteins, which are free from the limitations typically associated with milk proteins. The intrinsic characteristics of the protein composition in these samples provided more stable biological value indices.

These findings provided the rationale for further in vivo investigation of the effects of the developed composite ingredients on physiological status.

### 3.2. Feeding Behavior and Body Mass

**Figure 4:**
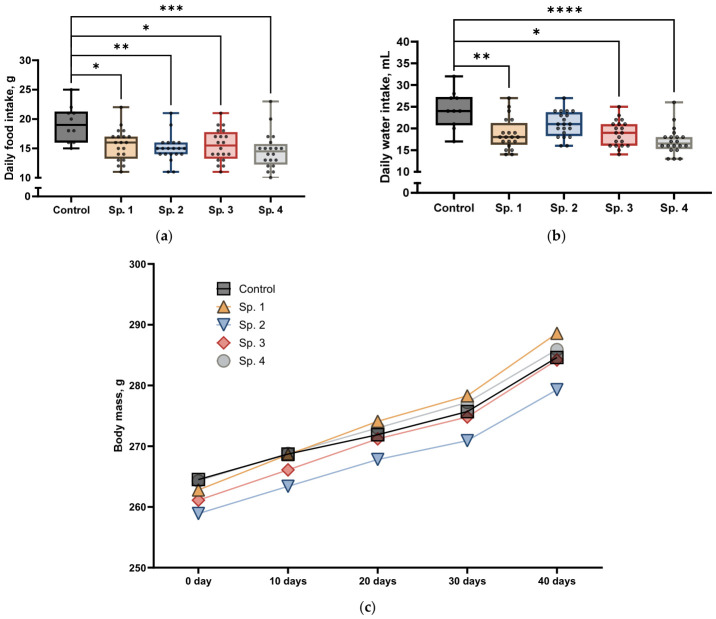
Feeding behavior: (**a**) Food intake (g/animal); (**b**) Water intake (mL/animal); (**c**) Body weight over time (g). Note: In panels (**a**,**b**), data are presented as box-and-whisker plots with individual data points overlaid. The box represents the interquartile range (25 th to 75 th percentiles), the horizontal line indicates the median, and the whiskers extend to minimum and maximum values. In panel (**c**), data are presented as mean values. Statistical comparisons were performed using Dunn’s test for non-parametric data. * *p* < 0.05, ** *p* < 0.01, *** *p* < 0.001, **** *p* < 0.0001 vs. Control. n = 20 per experimental group, n = 10 for Control.

Oral administration of high-protein samples for 40 days had a statistically significant effect on feeding behaviour in the animals (Figure 4). In all experimental groups, a statistically significant reduction in daily standard chow consumption was observed compared to the control group: by 19% (*p* < 0.01) in the Sample 1 group, by 22% (*p* < 0.01) in the Sample 2 group, by 19% (*p* < 0.01) in the Sample 3 group, and by 24% (*p* < 0.01) in the Sample 4 group. Similar changes were observed in water intake (Figure 4b).

Despite the reduction in food intake, body weight dynamics in all experimental groups did not differ significantly from the control group (Figure 4c), suggesting that nutritional balance was maintained through the administered supplements.

### 3.3. Endurance

**Figure 5:**
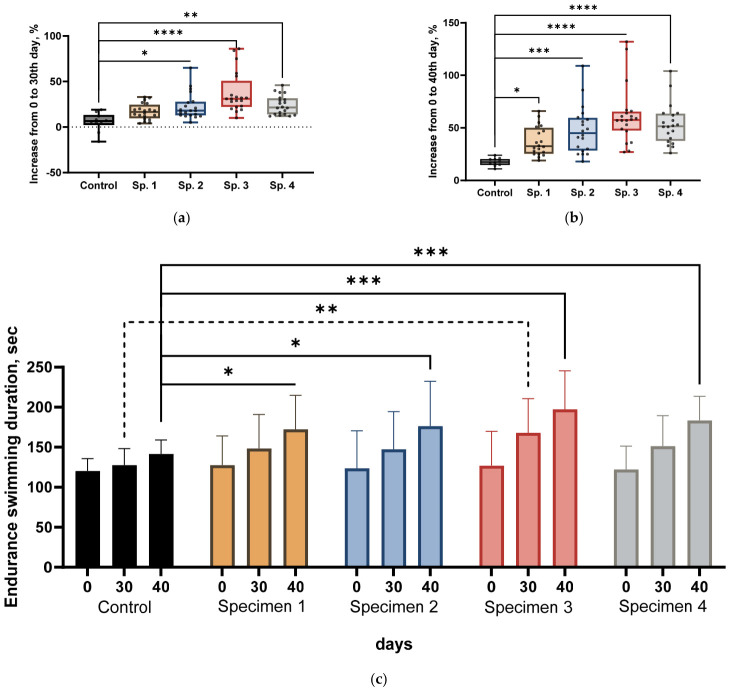
Endurance swimming: (**a**) Change in swimming time to exhaustion at day 30 relative to baseline; (**b**) Change in swimming time to exhaustion at day 40 relative to baseline; (**c**) Swimming time to exhaustion across all time points. Note: In panels (**a**,**b**), data are presented as box-and-whisker plots with individual data points overlaid. The box represents the interquartile range (25 th to 75 th percentiles), the horizontal line indicates the median, and the whiskers extend to minimum and maximum values. The dotted line in panel (**a**) indicates zero on the y-axis. In panel (**c**), data are presented as mean ± Standard deviation (SD, bar graph); dotted line represents the comparison groups at day 30 that differed significantly. Statistical comparisons: Dunn’s test for panels (**a**,**b**); two-way ANOVA followed by Dunnett’s post hoc test for panel (**c**). * *p* < 0.05, ** *p* < 0.01, *** *p* < 0.001, **** *p* < 0.0001 vs. Control. n = 20 per experimental group, n = 10 for Control.

Repeated-dose administration of the test samples improved physical endurance in the weighted forced swimming test. All experimental groups demonstrated a markedly greater positive change in weighted swimming duration at day 40 of the experiment (Figure 5c). At the interim time point—30 days of administration—a significant difference in weighted swimming duration (+32% in absolute values) relative to the control group was observed only in the Sample 3 group (*p* < 0.01). At day 40, a significant increase in swimming duration compared to the control was recorded in the Sample 3 group (+39%, *p* < 0.05) and the Sample 4 group (+30%, *p* < 0.05).

When analyzing the change in swimming duration relative to baseline (day 0), all experimental groups showed a statistically significant improvement (*p* < 0.05) compared to the control group (Figure 5a,b), indicating a pronounced positive effect of protein supplementation on endurance development.

### 3.4. Behavior

**Figure 6:**
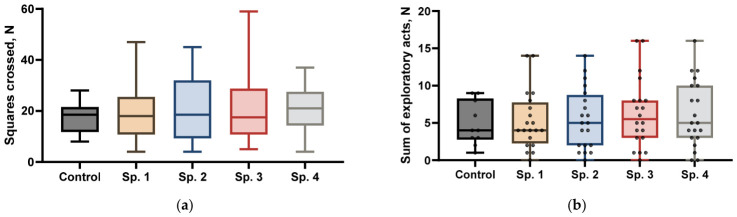

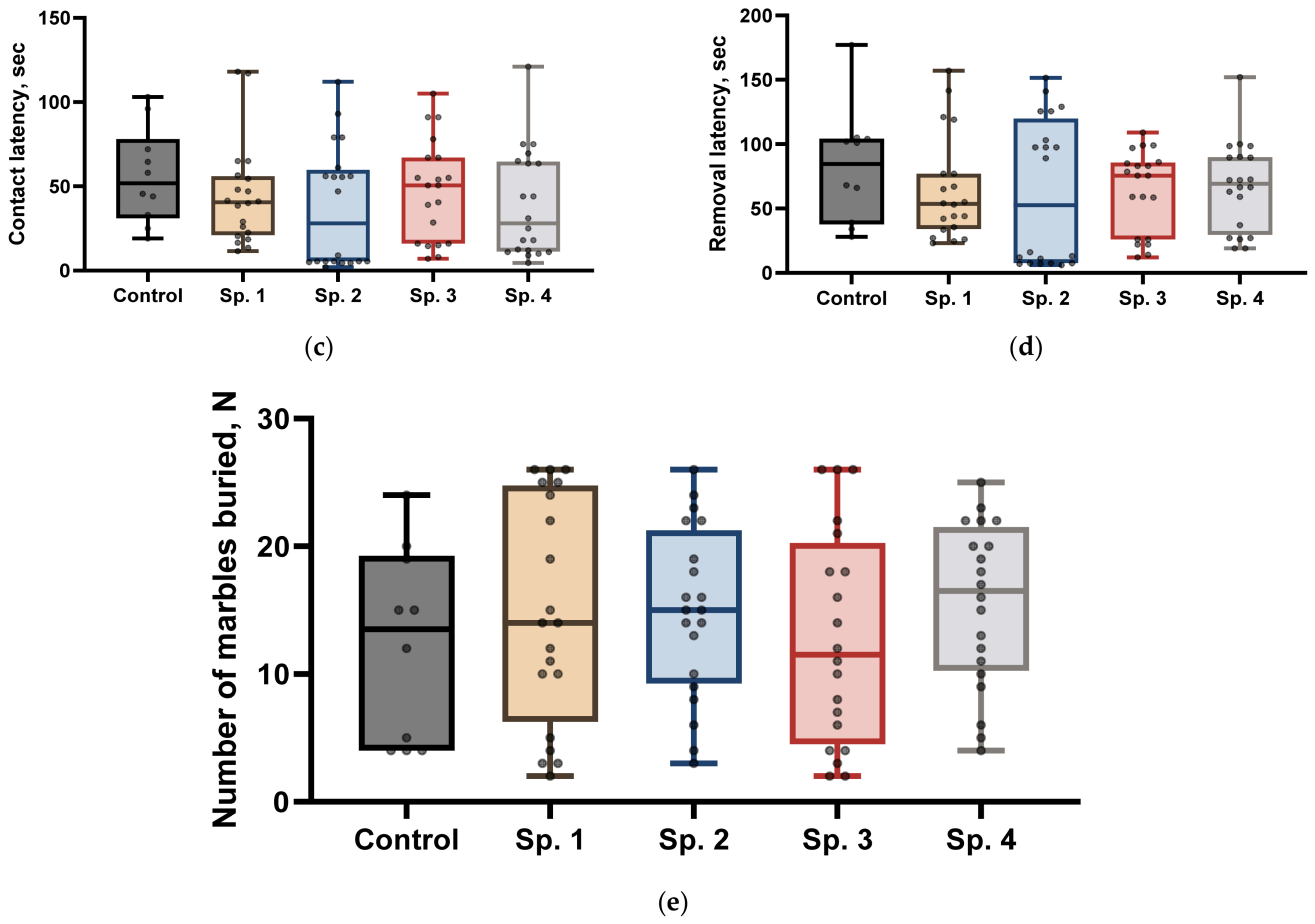
Behavioral tests: (**a**) Locomotor activity in the open field test; (**b**) Total exploratory activity; (**c**) Contact latency in the adhesive removal test; (**d**) Removal latency in the adhesive removal test; (**e**) Number of marbles buried in the marble burying test. Note: Data are presented as box-and-whisker plots with individual data points overlaid, except for panel (**a**), in which individual data points are omitted because the data followed a normal distribution. The box represents the interquartile range (25 th to 75 th percentiles), the horizontal line indicates the median, and the whiskers extend to the minimum and maximum values.

Assessment in the open field test revealed no adverse effects of the supplements on the psychoemotional state of the animals (Figure 6). No signs of anxiety-like behavior, suppression, or hyperexcitability were observed in any group. In the “Sample 3” and “Sample 4” groups, a trend toward increased locomotor activity (+26% and +19%, respectively) and exploratory activity (+29% and +27%, respectively) was noted; however, these differences did not reach statistical significance (*p* > 0.05). The results of the adhesive removal (Figure 6d) test and the marble burying test (Figure 6e) further confirmed the absence of impairments in sensorimotor function and compulsive defensive behavior across all experimental groups.

### 3.5. Biochemistry

**Figure 7:**
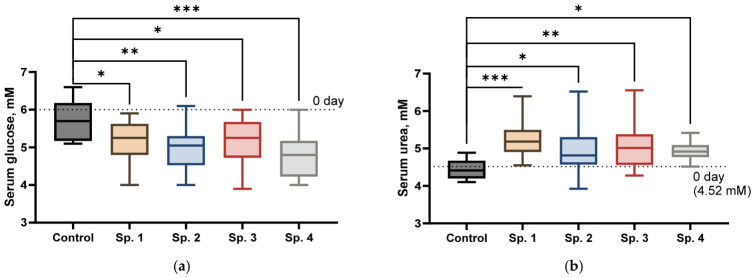

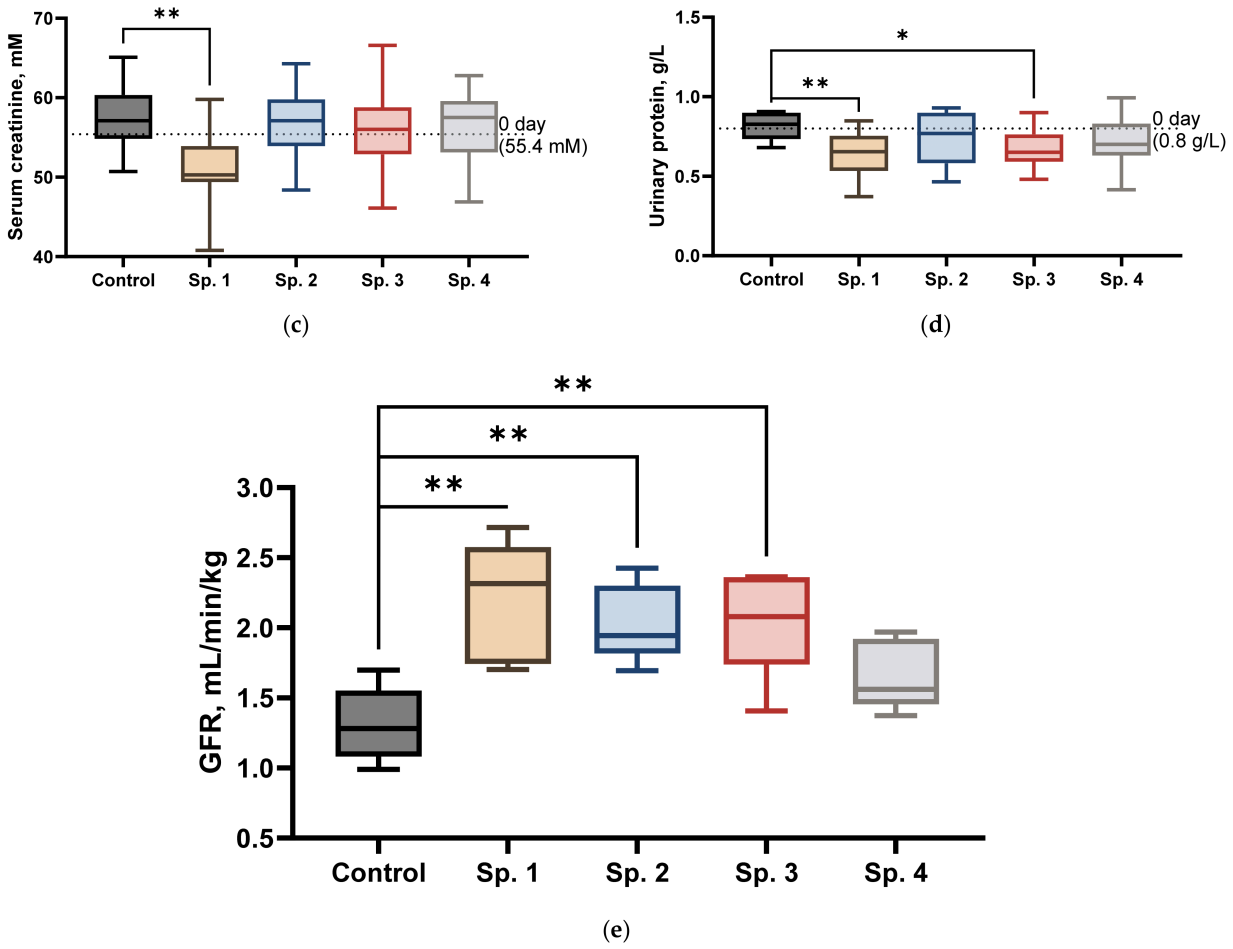
Biochemical parameters and renal function at day 40: (**a**) Blood glucose level (mmol/L); (**b**) Serum urea (mmol/L); (**c**) Serum creatinine (μmol/L); (**d**) Urinary protein (g/L); (**e**) Glomerular filtration rate (mL/min/kg). Note: Data are presented as box-and-whisker plots. The box represents the interquartile range (25 th to 75 th percentiles), the horizontal line indicates the median, and the whiskers extend to minimum and maximum values. The dotted lines in panels (**a–d**) show the average values on day 0 for all animals. Statistical comparisons were performed using Dunnett’s test. * *p* < 0.05, ** *p* < 0.01; *** p < 0.001 vs. Control. n = 20 per experimental group, n = 10 for Control (panels (**a**–**d**)); n = 5 per group for GFR (panel (**e**)).

Glucose metabolism: A statistically significant reduction in blood glucose levels was recorded in all experimental groups compared to the control: by 10% (*p* < 0.05) in the Sample 1 group, by 13% (*p* < 0.05) in the Sample 2 group, by 9% (*p* < 0.05) in the Sample 3 group, and by 16% (*p* < 0.05) in the Sample 4 group (Figure 7a).

Renal function: Supplementation was associated with a significant increase in serum urea levels in all experimental groups (from +9% to +16%, *p* < 0.05), reflecting an increased nitrogen load (Figure 7). In the Sample 1 group, a statistically significant reduction in serum creatinine was recorded (−11%, *p* < 0.05, Figure 7c). A favourable effect on urinary protein excretion was also observed: in the Sample 1 and Sample 3 groups, proteinuria was significantly lower than in the control by 19% (*p* < 0.05) and 16% (*p* < 0.05), respectively (Figure 7d).

GFR in the control group fed a standard diet was consistent with values reported for healthy animals. In all experimental groups, a trend toward increased GFR was observed following completion of the administration course. In the groups receiving test samples 1, 2, and 3, GFR was significantly higher (*p* < 0.05) than in the control group (Figure 7e). These findings indicate a pronounced improvement in glomerular filtration in the experimental animals following repeated-dose administration of the test samples.

Overall assessment of the biochemical markers revealed no signs of renal impairment; the observed changes were interpreted as an adaptive response to the high-protein diet.

## 4. Discussion

The present findings, demonstrating a significant reduction in standard chow consumption alongside stable body weight, are consistent with studies in which a high-protein diet promoted rapid satiety and improved metabolism (i.e., more efficient nutrient utilisation). For example, Campos de Macêdo M.R. et al. (2023) reported that whey protein supplementation (4 and 6 g/kg/day) over 12 weeks dose-dependently reduced food intake and body weight in Wistar rats relative to controls; body weight increased steadily (unlike our study, lagging slightly behind controls), while food intake remained comparable to week-one levels for the first 6 weeks before declining gradually. No physical activity tests were included in that study design [20]. De Almeida P.C. et al. (2020) [21] combined a high-protein diet (based on whey protein isolate) with resistance training in one group, while the other group received the diet without training. The authors reported a significant improvement in physical performance in both groups. Additionally, changes in the fat-to-muscle tissue ratio were observed, more pronounced in the trained group. A statistically non-significant reduction in food intake was also noted. The authors suggested that a high-protein diet exerts a pronounced effect on metabolism, promoting muscle tissue growth and fat mass reduction even in the absence of physical exercise. Body weight across all high-protein diet series was non-significantly lower than in the respective control groups [21], in contrast to the findings of da Rosa Lima et al. (2018), in which combined resistance training and a high-protein diet (high-casein) over 8 weeks resulted in a non-significantly higher final body weight compared to exercise alone, while the diet without exercise had no effect on body weight over the same period [22].

In this 40-day study, the loaded swimming test was performed three times, with two repeated assessments conducted during the period of protein supplementation, which may have influenced body weight gain dynamics. Nevertheless, body weight increased steadily during the first 30 days in the experimental groups, similarly to the control group, suggesting compensation for nutrients not sufficiently supplied by the standard diet. During the subsequent 10-day period following the second loaded swimming test (day 30), a modest acceleration in body weight gain was observed in the experimental groups, both relative to the preceding 10-day intervals and in comparison, with the control group. Thus, the observed standard chow intake and body weight dynamics during the course administration of the high-protein test materials are consistent with previously published studies using broadly similar experimental designs.

The present findings on chow intake and body weight dynamics are consistent with published evidence on the effects of high-protein diets on feeding behavior and body composition. In their review, Journel et al. (2012) concluded that dietary proteins play a central role in satiety, with long-term high-protein feeding reducing food intake, body weight, and adipose tissue mass in both rats and humans [23]. Experimental studies have additionally identified physical activity as an important [22], and in some cases desirable [21], factor for modifying feeding behavior and correcting metabolic disturbances. Following ingestion of a high-protein diet, enteroendocrine cells release anorexigenic hormones, primarily cholecystokinin, as well as glucagon-like peptide-1 and peptide YY, which stimulate vagal afferents and transmit satiety-related signals to the nucleus tractus solitarius and the arcuate nucleus, while also modulating mesolimbic reward pathways [23]. Protein composition itself also appears to be a key determinant of dietary efficacy. Pichon et al. (2008) demonstrated that whey proteins and β-lactoglobulin within high-protein diets more effectively reduced chow intake and body weight gain in Wistar rats than whole milk protein, an effect attributed to enhanced satiety signaling [24].

The 29–39% improvement in swimming endurance observed in the present study indicates a substantial enhancement of physical capacity attributable to the administered test samples. The improvement in physical performance may be related to enhanced muscle protein synthesis, more efficient recovery from exercise-induced muscle damage, and optimisation of energy metabolism. These findings are consistent with those of Ren et al. (2017), in which 7 weeks of whey protein supplementation in Wistar rats increased weighted (10% body weight) swimming time to exhaustion by approximately 25%; however, when soy protein was added to the whey protein, this figure increased to 50% [25]. A more pronounced effect on physical performance was reported in the study by De Almeida et al. (2020), in which resistance training using the maximum load test (MLT) was conducted over 6 weeks (3 sessions per week): MLT performance in the protein-supplemented experimental group exceeded that of the control group by 106%. However, in a parallel series of untrained rats, the protein-supplemented group lifted 126% more weight than the corresponding control group (no training) [21], highlighting the significance of the training effect per se, even under a standard diet, which was also observed in the present study.

The improvement in physical endurance observed across all experimental groups warrants consideration of collagen hydrolysate-specific mechanisms that may operate independently of total protein intake. Unlike conventional protein supplements, enzymatic hydrolysates of collagen yield a characteristic pool of bioactive di- and tripeptides—primarily prolyl-hydroxyproline (Pro-Hyp) and hydroxyprolyl-glycine (Hyp-Gly)—that are detectable in peripheral blood following oral ingestion, indicating systemic bioavailability of intact peptide forms [26]. These peptides are not found in appreciable quantities in whey or other standard protein sources and may therefore confer effects distinct from general amino acid supplementation.

With respect to physical endurance, collagen-derived peptides have been shown to activate the AMPK/PGC-1α/NRF1/TFAM signalling pathway in skeletal muscle, promoting mitochondrial biogenesis, increasing ATP production and muscle glycogen content, enriching slow-twitch oxidative muscle fibres, and extending exhaustive swimming duration in rodent models. Concurrently, activation of the NRF2/HO-1 antioxidant pathway reduces exercise-induced reactive oxygen species accumulation and protects mitochondrial integrity [27]. These mechanisms provide a plausible mechanistic basis for the improvements in weighted swimming endurance observed in the present study, particularly the most pronounced effect in the Sample 3 group, whose composition—combining bovine collagen hydrolysate with meat trim hydrolysate, porcine blood plasma proteins, and an api-component—may have yielded a more diverse bioactive peptide profile.

At the molecular level, the collagen-derived dipeptide Hyp-Gly has been shown to activate the PI3K/Akt/mTOR signalling pathway in C2C12 myoblasts, stimulating myogenic differentiation and myotube hypertrophy independently of leucine [28]. Pro-Hyp has similarly been reported to phosphorylate Akt and protect myotubes from atrophy. Although these findings derive from in vitro models and require confirmation in vivo, they suggest that collagen peptides may contribute to muscle anabolic signalling through mechanisms not dependent on BCAA content—an important distinction given that the observed endurance improvements occurred without an overall increase in total protein intake. Additionally, Pro-Hyp has been shown to directly upregulate PGC-1α gene expression through a peptide-responsive element in the PGC-1α promoter, providing a molecular link between collagen peptide intake and mitochondrial biogenesis [29].

The significant reduction in blood glucose levels observed in the present study may be an obvious consequence of reduced food intake associated with repeated-dose administration of high-protein samples, as noted in several studies with a similar design [21,30]. However, some authors have emphasised the critical role of physical exercise in such study designs, as exercise can increase tissue sensitivity to insulin—primarily in muscle tissue—and improve glycaemic control, accompanied by a reduction in plasma concentrations of pro-inflammatory cytokines [30]. Baum et al. (2006) reported that under a high-protein, low-carbohydrate diet, postprandial fluctuations in blood glucose and insulin were attenuated, and glucagon levels were significantly lower than in the control group receiving a standard diet [31]. Stepien et al. (2006) observed a reduction in blood insulin levels following 2 weeks of a high-protein diet, possibly reflecting a feedback response to increased insulin sensitivity in insulin-dependent tissues [32]. Dugardin et al. (2022) provided evidence supporting the hypothesis that certain peptides and amino acids influence glucose absorption, in part through regulation of glucose transporter 2 (GLUT2) expression [33].

A similar effect of a high-protein diet on glycaemic levels has been reported under conditions of impaired carbohydrate metabolism. In a rat model of experimental obesity, a high-protein diet selectively reduced fat mass and improved glucose tolerance [34]. Gannon et al. (2003) [35] reported a 40% reduction in the 24 h blood glucose area under the curve (AUC) in individuals with type 2 diabetes. However, the authors leave open the question of the safety of this approach in patients for whom the kidneys are among the target organs of the underlying disease [35]. Thus, the reduction in blood glucose levels observed in the experimental groups following repeated-dose administration of high-protein test samples confirms the ability of such diets to normalise carbohydrate metabolism, which is well documented in the literature. Among the possible mechanisms by which a high-protein diet influences carbohydrate metabolism, a key role may be played by changes in feeding behaviour: reduced standard food consumption via central mechanisms [23], slowed carbohydrate absorption [33], increased insulin sensitivity in insulin-dependent tissues (primarily muscle) [30,36,37], and reduced levels of the counter-regulatory hormone glucagon [31].

Regarding glucose metabolism, the reduction in blood glucose levels observed across all experimental groups is consistent with a GLP-1-mediated mechanism specific to collagen hydrolysates. Intestinal enteroendocrine L-cells express receptors sensitive to specific peptide sequences, and collagen hydrolysates have been shown to stimulate GLP-1 secretion both in vitro and in vivo, resulting in improved postprandial glucose tolerance in rodent models and, in a proof-of-concept human study, at a dose of 5 g in normoglycaemic and prediabetic participants [38]. This mechanism—involving enhanced incretin signalling, slowed gastric emptying, and increased insulin secretion—is distinct from the general metabolic effects of high-protein diets and supports the interpretation that the glycaemic improvements observed in the present study reflect, at least in part, a collagen peptide-specific biological activity. Importantly, GLP-1 secretion was not measured in the present study, and direct confirmation of this pathway requires dedicated mechanistic investigation.

Taken together, the available evidence supports the hypothesis that the physiological effects observed in the present study are not solely attributable to increased protein intake or caloric restriction, but reflect the specific bioactivity of collagen-derived peptides operating through distinct signalling pathways in skeletal muscle and intestinal endocrine cells.

The absence of negative behavioural effects and anxiogenic effects, together with a moderate increase in locomotor and exploratory activity, represent important safety observations. Results of the open field test, adhesive removal test, and marble burying test indicate that repeated-dose administration of the high-protein samples did not induce any motor, sensorimotor, or anxiety-depressive disturbances. A moderate increase in locomotor and exploratory activity, presumably associated with a mild anxiolytic effect of the test samples, has also been reported in the literature. The elevated locomotor activity observed in the present study coincides with improved exercise tolerance, suggesting that a high-protein diet may enhance physical activity, possibly through stimulation of muscle protein synthesis or shifts in energy metabolism. Although studies with an identical design are not available in the literature, several studies conducted in aged animals with longer periods of high-protein diet administration have reported increased locomotor activity and reduced anxiety levels in the open field test [11,36,39,40,41].

According to the results of the present study, no signs of renal function deterioration were detected following 40 days of administration of the high-protein test samples. The elevation in urea levels reflects enhanced protein turnover and increased nitrogen load, which is an expected consequence of augmented protein catabolism [42]. The reduction in serum creatinine (in the Sample 1 group) and proteinuria (across all groups) may indicate a compensatory improvement in tubular reabsorption or reduced glomerular permeability, which is characteristic of adaptation to a high-protein diet, as has also been reported in several studies [22,36,42]. The observed changes—elevated serum urea alongside reduced serum creatinine and urinary protein—are indicative of a positive nitrogen balance and metabolic adaptation to a high-protein diet, accompanied by activation of enzymatic nitrogen utilisation pathways. The absence of signs of renal functional impairment under a substantial protein load suggests the engagement of compensatory physiological mechanisms that redirect protein metabolism toward efficient urea synthesis while maintaining tubular reabsorption within the physiological range.

Therefore, when interpreting the present findings, it should be noted that the observed improvement in biochemical markers of renal function may reflect not only genuine nephroprotection but also a phase of functional compensation characteristic of the early period of adaptation to a high-protein diet. According to published data, at later time points (8–12 weeks) rodents may develop morphological signs of nephron overload despite normal creatinine and proteinuria levels at earlier stages [42,43,44,45]. Furthermore, several studies have reported that physical exercise combined with a high-protein diet exerts a nephroprotective effect and contributes to attenuation of the biochemical shifts in markers of renal hyperfiltration [22,30].

Taken together, the present findings indicate that repeated-dose administration of the test samples to experimental animals produces favourable metabolic changes in healthy adult rats: physical performance was substantially improved, food intake was reduced without affecting body weight—with nutrient deficits compensated by the supplements—carbohydrate metabolism was normalised, renal function markers were improved, and no adverse effects on the psychoemotional status of the animals were observed. However, the long-term sustainability of these favourable effects warrants further investigation, particularly with regard to the transition point between beneficial adaptation and potential decompensation of renal function. The normalising effect of physical exercise on renal function markers—even those shifted in a favourable direction—reported in more complex studies of similar design using comparable formulations [21,22,30] suggests that the test samples hold potential not only as agents that improve exercise tolerance, but also in terms of their safety profile, provided that their use is appropriately justified with respect to target population, conditions of administration, dose optimisation, and duration of intake.

The administered dose of 3000 mg/kg/day constituted approximately 5% of total daily food intake by mass, based on a mean daily consumption of approximately 15 g in control animals. Based on the typical nutritional composition of standard rodent diet per GOST R 51849-2001 (approximately 23% protein, 5% fat, 50% carbohydrates, 3.4 kcal/g), total daily protein intake in experimental groups remained comparable to controls: animals receiving the supplement obtained approximately 3.40 g protein/day (2.74 g from standard diet plus 0.66 g from the supplement, given a protein content of ~88% in the administered powder) versus 3.45 g/day in the control group. This indicates that the observed physiological effects were not driven by an overall increase in protein intake, but rather reflect a qualitative shift in protein source toward collagen hydrolysate-based complexes. Total caloric intake in the experimental groups was modestly reduced (approximately 43 vs. 51 kcal/day), and a contribution of mild caloric restriction to the observed improvements in glucose metabolism cannot be entirely excluded. However, caloric restriction alone is not expected to improve physical endurance—and may in fact impair it—making it unlikely to account for the significant gains in weighted swimming performance observed across all experimental groups.

Extrapolation of the effective dose to humans using the body surface area conversion factor recommended by the FDA (rat-to-human scaling factor of 6.2) yields an equivalent human dose of approximately 484 mg/kg/day, corresponding to ~34 g of supplement (~30 g protein) per day for a 70 kg individual. This range is consistent with doses employed in clinical trials of collagen peptide supplementation in humans: improvements in running endurance performance have been reported with 15 g/day in moderately trained men [46] and women [47] undergoing concurrent training, and favourable effects on muscle-tendon mechanical properties and explosive strength were demonstrated with 10 g/day in sedentary males [48]. Regarding glucose metabolism, a specific collagen hydrolysate reduced the postprandial glucose response at a dose of 5 g in healthy and prediabetic humans, with the proposed mechanism involving stimulation of intestinal GLP-1 secretion [38]. Collectively, these findings suggest that the physiological effects observed in the present study may be achievable in humans at practically feasible supplementation levels, supporting the translational potential of the tested ingredients. Confirmation of efficacy and safety at this dose range in human populations remains a subject for future clinical investigation.

### Limitations

Several limitations should be acknowledged when interpreting our findings. First, the 40-day study duration, while sufficient to demonstrate adaptive physiological responses, is relatively short for assessing long-term renal safety. Morphological changes such as focal glomerulosclerosis in rats fed high-protein diets typically emerge after 20–36 weeks [49], and significant renal fibrosis requires 8+ months of exposure [45]. Our observed improvements in biochemical markers (reduced proteinuria, increased GFR) may represent early adaptive responses rather than sustained protection, as the transition from beneficial hyperfiltration to maladaptive glomerular injury can occur with prolonged exposure [45].

Second, we did not perform histological examination of renal tissue. While our biochemical markers suggest preserved kidney function, subclinical morphological changes (glomerular hypertrophy, early fibrotic changes) can precede functional decline and may not be detectable through standard biomarkers alone [42,45]. Future studies should include renal histopathology to confirm the absence of structural damage.

Third, only male rats were studied, limiting generalizability to females, who may exhibit different metabolic and renal responses to high-protein intake. Fourth, we tested a single dose (3000 mg/kg/day), precluding establishment of a dose–response relationship or determination of an optimal dose.

Fifth, GFR measurements were performed in 5 animals per group due to the technical complexity of 24 h metabolic cage collections and creatinine clearance measurements. While this sample size is smaller than the main experimental cohort (n = 20), the data demonstrated normal distribution (Shapiro–Wilk test) and sufficient statistical power to detect significant differences between groups (specimens 1, 2 and 3), with all experimental groups showing significantly elevated GFR compared to controls (*p* < 0.05).

Finally, the forced swimming test protocol, while demonstrating improved endurance, represents a form of exercise intervention that may have confounded our assessment of protein supplementation effects alone. Exercise is known to mitigate metabolic acidosis and renal stress induced by high-protein diets [42,45], making it difficult to isolate the direct effects of protein complexes from potential protective effects of physical activity.

A further limitation concerns the control design. The absence of an isocaloric, isonitrogenous control group precludes definitive attribution of the observed effects to the collagen hydrolysate-based composition specifically, as opposed to a general response to the modest caloric deficit (~43 vs ~51 kcal/day) or supplemental protein intake. Additionally, the multi-component nature of the experimental samples, while reflective of real-world functional ingredient formulations, limits the ability to isolate the contribution of individual components. Disentangling the specific roles of each constituent remains an important objective for future studies.

Despite these limitations, our findings provide initial evidence of favorable short-term metabolic and renal adaptation to collagen hydrolysate-based protein complexes in healthy adult rats.

## 5. Conclusions

Based on the findings of the present study, and in accordance with its aims and objectives, the following conclusions are drawn:

1. Effects on physical condition: Repeated-dose administration of animal-derived high-protein ingredients over 40 days exerted a pronounced positive effect on the physical condition of the laboratory animals, manifested as a significant improvement in physical endurance (numerically highest effect observed with Sample 3) and normalisation of carbohydrate metabolism (reduction in blood glucose levels; numerically highest effect observed with Sample 4).
2. Effects on feeding behaviour: All test samples significantly altered feeding behaviour, reducing standard chow consumption while maintaining normal body weight gain dynamics, indicating effective compensation of nutritional balance by the administered supplements.
3. Effects on psychological status and safety: The test ingredients exerted no adverse effects on psychological status, sensorimotor function, or anxiety levels in the animals, confirming a favourable safety profile within the scope of the present experiment.
4. Effects on renal function: No signs of renal function deterioration were detected under conditions of elevated protein load. The observed changes—elevated urea concentration alongside reduced or stable creatinine levels and proteinuria—are interpreted as an adaptive physiological response rather than a pathological disturbance.

The present study allows us to conclude that animal-derived high-protein ingredients are both effective and safe under conditions of 40-day administration. They demonstrate a pronounced positive effect on key physical health parameters—endurance and glucose metabolism—with no adverse effects on psychological and emotional wellbeing or neurological status.

Among the experimental groups, Sample 3 showed the numerically highest mean swimming duration at day 40 and the largest effect size relative to control, while Sample 4 demonstrated the numerically lowest values for standard diet consumption and blood glucose. The findings regarding renal function adaptation to an elevated protein load are encouraging and suggest a robust compensatory capacity of the organism.

Thus, the results of the present study support the potential of these ingredients as specialised food products for improving physical fitness and metabolic health. To confirm the long-term safety and sustainability of the observed beneficial effects, studies of longer duration and in additional animal models are recommended.

## Figures and Tables

**Table 1:** Functional and technological properties of the developed complex ingredients.

Parameter	Specimen 1	Specimen 2	Specimen 3	Specimen 4
Water-binding capacity, % of total moisture	93.33 ± 2.35	93.33 ± 2.33	95.31 ± 2.35	98.06 ± 2.42
Water-holding capacity, % of dry matter	73.32 ± 9.21	73.36 ± 9.21	73.78 ± 9.23	71.18 ± 9.21
Fat-holding capacity, % of dry matter	21.41 ± 1.21	15.41 ± 1.21	20.41 ± 1.25	23.11 ± 1.11
Plasticity, ×10^−2^ cm^2^/g	10.49 ± 0.26	10.49 ± 0.26	10.49 ± 0.26	10.30 ± 0.25
Yield stress, Pa	30.76 ± 5.71	30.77 ± 5.71	31.76 ± 5.72	30.76 ± 5.95

**Table 2:** Chemical and nutrient composition of the developed complex ingredients.

Parameter	Specimen 1	Specimen 2	Specimen 3	Specimen 4
Moisture content, g/100 g	6.00 ± 0.32	6.00 ± 0.31	6.00 ± 0.35	6.00 ± 0.42
Protein content, g/100 g	89.00 ± 0.32	88.00 ± 0.33	89.00 ± 0.23	88.00 ± 0.21
Fat content, g/100 g	4.00 ± 0.35	5.00 ± 0.33	4.00 ± 1.25	5.00 ± 1.11
Carbohydrate content, g/100 g	LLOQ	LLOQ	LLOQ	LLOQ
Vitamin C, mg/100 g	-	-	86.3 ± 1.03	86.5 ± 1.03
Iron Fe^3+^, μg/100 g	-	-	11.9 ± 2.55	11.8 ± 2.55
Vitamin K, mg/100 g	-	-	800.1 ± 11.15	800.6 ± 11.15
In vitro digestibility, mg tyrosine/g protein				
Pepsin phase	6.33 ± 0.11	5.99 ± 0.11	6.59 ± 0.17	6.36 ± 0.16
Trypsin phase	10.16 ± 0.25	10.16 ± 0.25	10.46 ± 0.25	10.21 ± 0.24
Total	16.49 ± 0.43	16.15 ± 0.43	17.15 ± 0.43	16.57 ± 0.40

Note: LLOQ—Lower limit of quantification.

**Table 3:** Biological value indicators of complex ingredients.

Amino Acid	Specimens 1, 2		Specimens 3, 4		FAO/WHO Reference Pattern	
	Content	Score, %	Content	Score, %	Content	Score, %
Essential amino acids						
Isoleucine	5.68 ± 0.13	142.00	5.93 ± 0.18	148.25	4.00	100
Leucine	8.12 ± 0.20	116.00	8.37 ± 0.23	119.57	7.00	100
Lysine	9.28 ± 0.23	168.73	8.87 ± 0.20	161.27	5.50	100
Methionine and Cystine	3.72 ± 0.09	106.28	3.67 ± 0.10	104.86	3.50	100
Phenylalanine and L-Tyrosine	7.24 ± 0.20	120.67	7.68 ± 0.18	128.00	6.00	100
Threonine	5.73 ± 0.14	143.25	5.56 ± 0.09	139.00	4.00	100
Tryptophan	1.32 ± 0.04	132.00	1.15 ± 0.03	115.00	1.00	100
Valine	5.65 ± 0.14	113.00	5.76 ± 0.17	115.20	5.00	100
Biological value indices						
Biological value, %	75.18		76.82		100	
R_C_, relative units	0.80		0.82		R_C_→1	
α, g/100 g reference protein	8.40		8.81		α→0	

Note: R_C_—protein utility coefficient; α—comparable redundancy coefficient.

## Data Availability

The data supporting the findings of this study have been deposited in the Figshare repository and are openly available at https://doi.org/10.6084/m9.figshare.31941006, accessed on 5 April 2026). The dataset includes all raw data necessary to reproduce the results presented in this article.

## Abbreviations

The following abbreviations are used in this manuscript:

GFR	Glomerular filtration rate
LLOQ	Lower limit of quantification
ANOVA	Analysis of variance
MLT	Maximum load test

## Appendix B

*Water-holding capacity (method of Lipatov Jr.)*

Twenty to twenty-five grams of the sample are placed into pre-weighed jars. The jars are labelled, sealed with lids, and heated until the temperature at the centre of the jar reaches 70 ± 2 °C. The jars are then cooled, the lids removed, and the released broth drained into a container of known mass and weighed; the jars with the remaining contents are also weighed. The moisture content of the collected broth is determined.

WHC = X_0_m_0_ − {m_n_ − m_k_ + m_b + m_b·X_v}/[m_0_(1 − X_0_)] · 100, %, (A1)

where WHC—water-holding capacity, % of dry matter; m_b—mass of released broth, g; X_v—moisture content of released broth, fraction; m_n_—initial mass of jar with sample, g; m_k_—mass of jar with sample after heat treatment and broth separation, g; m_0_—mass of sample placed in jar, g; X_0_—moisture content of sample, fraction.

*Fat-holding capacity (method of Lipatov Jr.)*

Ten to fifteen grams of the sample are placed into pre-weighed jars with the addition of vegetable oil at 30% of the sample mass. The jars are tightly sealed, labelled, and subjected to heat treatment in a drying oven at 75–80 °C until the temperature at the centre of the sample reaches 70 ± 2 °C. The jars are then cooled and the broth drained; the broth is subsequently heated at 60 °C in a water bath for 15–20 min to allow separation of broth from fat.

FHC = [0.3·m_0_ − 0.975·V_f]/[(1 − X_0_)·m_0_ − 0.3·m_0_] · 100, (A2)

where m_0_—sample mass before heat treatment, g; V_f—volume of separated fat from broth, mL; X_0_—moisture content of sample, %; 0.3 and 0.975—coefficients.

*In vitro protein digestibility (Pokrovsky–Ertanov method)*

Protein digestibility was assessed by sequential exposure of sample proteins to a protease system consisting of pepsin and trypsin using a modified apparatus (MGUPP, Russian Federation). Accumulated enzymatic hydrolysis products were quantified by the Lowry colorimetric reaction on a spectrophotometer and expressed as mg tyrosine per g protein.
